# Silhouette scores for assessment of SNP genotype clusters

**DOI:** 10.1186/1471-2164-6-35

**Published:** 2005-03-10

**Authors:** Lovisa Lovmar, Annika Ahlford, Mats Jonsson, Ann-Christine Syvänen

**Affiliations:** 1 Molecular Medicine, Department of Medical Sciences, Uppsala University, Uppsala, Sweden

## Abstract

**Background:**

High-throughput genotyping of single nucleotide polymorphisms (SNPs) generates large amounts of data. In many SNP genotyping assays, the genotype assignment is based on scatter plots of signals corresponding to the two SNP alleles. In a robust assay the three clusters that define the genotypes are well separated and the distances between the data points within a cluster are short. "Silhouettes" is a graphical aid for interpretation and validation of data clusters that provides a measure of how well a data point was classified when it was assigned to a cluster. Thus "Silhouettes" can potentially be used as a quality measure for SNP genotyping results and for objective comparison of the performance of SNP assays at different circumstances.

**Results:**

We created a program (ClusterA) for calculating "Silhouette scores", and applied it to assess the quality of SNP genotype clusters obtained by single nucleotide primer extension ("minisequencing") in the Tag-microarray format. A Silhouette score condenses the quality of the genotype assignment for each SNP assay into a single numeric value, which ranges from 1.0, when the genotype assignment is unequivocal, down to -1.0, when the genotype assignment has been arbitrary. In the present study we applied Silhouette scores to compare the performance of four DNA polymerases in our minisequencing system by analyzing 26 SNPs in both DNA polarities in 16 DNA samples. We found Silhouettes to provide a relevant measure for the quality of SNP assays at different reaction conditions, illustrated by the four DNA polymerases here. According to our result, the genotypes can be unequivocally assigned without manual inspection when the Silhouette score for a SNP assay is > 0.65. All four DNA polymerases performed satisfactorily in our Tag-array minisequencing system.

**Conclusion:**

"Silhouette scores" for assessing the quality of SNP genotyping clusters is convenient for evaluating the quality of SNP genotype assignment, and provides an objective, numeric measure for comparing the performance of SNP assays. The program we created for calculating Silhouette scores is freely available, and can be used for quality assessment of the results from all genotyping systems, where the genotypes are assigned by cluster analysis using scatter plots.

## Background

High-throughput single nucleotide polymorphism (SNP) genotyping assays generate large amounts of data, which usually is presented as scatter plots of signals corresponding to the two SNP alleles. A robust SNP genotyping assay is characterized by large distances between the three clusters that define the genotypes and small distances between the data points within each cluster. Numeric quality measures for the scatter plots would allow objective and automatic assessment of the success of a SNP assay.

"Silhouettes" were introduced in 1987 as a general graphical aid for interpretation and validation of cluster analysis [1]. In a Silhouettes calculation, the distance from each data point in a cluster to all other data points within the same cluster and to all data points in the closest cluster are determined. Thus Silhouettes provides a measure of how well a data point was classified when it was assigned to a cluster by according to both the tightness of the clusters and the separation between them. This feature renders Silhouettes potentially well suited for assessing cluster quality in SNP genotyping methods. In high-throughput SNP genotyping, Silhouettes could be used for assessing the quality of automatic genotype assignment by alerting the operator if the quality of the genotype clusters fall below a certain limit. During assay development and optimization, Silhouettes could be used to compare the performance of a genotyping assay at different reaction conditions. It could also be applied for comparing the robustness of different SNP genotyping technologies.

In this study we created a program (ClusterA) to calculate numeric Silhouettes for assessing the quality of genotype clusters obtained in SNP genotyping assays. We show the utility of Silhouettes and the program by applying it to our "in-house" developed four-color fluorescence minisequencing system for SNP genotyping in a microarray format [2]. Single nucleotide primer extension ("minisequencing") is the reaction principle underlying several of the commonly used systems for genotyping single nucleotide polymorphisms (SNPs) [3-8]. In minisequencing a DNA polymerase is employed to specifically extend a detection primer designed to anneal directly adjacent to the SNP position in the complementary DNA strand with a single labelled nucleotide analogue. The DNA polymerase is the most important factor that determines the efficiency and specificity of the primer extension reaction, irrespectively of the assay format. We used Silhouettes to compare the performance of three new commercially available DNA polymerases to the ThermoSequenase DNA polymerase, which is routinely used in minisequencing assays in many laboratories, including our own. We found Silhouettes to provide a relevant measure, in addition to signal-to-noise ratios and genotyping success, for selecting the most favourable enzyme for our assay.

## Results and Discussion

**Figure 1:**
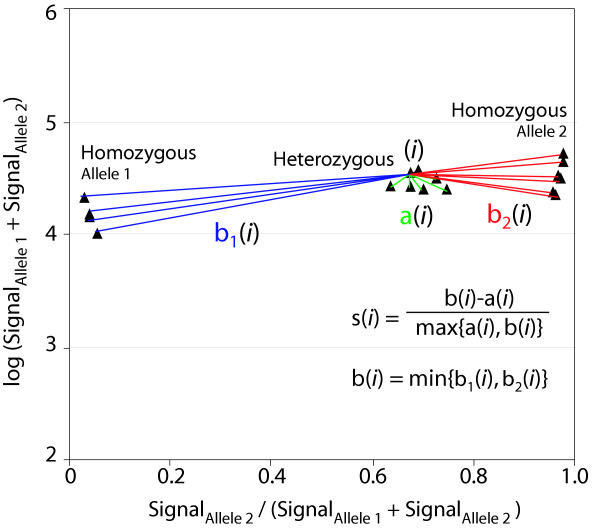
**Principle for Silhouette scores. **Principle for quality assessment of genotyping clusters using Silhouette scores, illustrated for one data point (i). The SNP genotypes have been assigned based on cluster formation in scatter plots with the signal intensity fraction on the x-axis and the logarithm of the signals from both alleles on the y-axis. For each data point (i) in the scatter plot, the Silhouette s(i) is calculated by the formula in the figure, where a(i) is the average distance from i to all data points in the same genotype cluster (green lines), and b(i) is the average distances from i to all data points in the cluster closest to the data point, either b_1_(i) (blue lines) or b_2_(i) (red lines) [1]. Max and min in the formula denote the largest or smallest of the measures in the brackets. The "average silhouette width" is calculated by calculating the mean of all s(i) for each genotype cluster and the "Silhouette score" for the whole scatter plot (SNP assay) is obtained by taking the mean of the average silhouette width for all clusters.

We created a program, denoted ClusterA, for calculating numeric "Silhouettes" for clustered data, such as for example the three clusters of signal ratios commonly obtained in SNP genotyping assays. Figure 1 illustrates the Silhouette calculation for one data point in a typical scatter plot obtained in a SNP genotyping assays. A Silhouette close to 1.0 is obtained when the average distance from a data point to the other data points within its own cluster is smaller than the average distances to all data points in the closest cluster. A Silhouette close to zero indicates that the data-point could equally well have been assigned to the neighbouring cluster. A negative Silhouette is obtained when the cluster assignment has been arbitrary, and the data point is actually closer to the neighbouring cluster than to the other data points within its own cluster [1]. The mean value from the Silhouette calculations for all data points in each cluster yield an "average Silhouette width" for the cluster.

**Figure 2:**
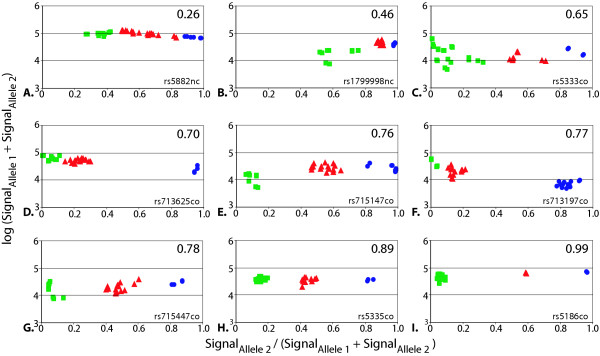
**Examples of Silhouette scores. **Examples of genotype clusters from nine SNP assays, each with the results from 16 samples genotyped in duplicate using Tag-array minisequencing with the calculated Silhouette scores shown in the right hand upper corner of each panel. The blue circles represent homozygotes for allele 2, the red triangles are heterozygotes and the green squares are homozygotes for allele 1. The SNPs are denoted by their dbSNP identification number, and the DNA polarities analyzed are indicated by "cod" or "nc".

Here, we applied ClusterA to calculate "Silhouettes" for comparing the quality of the genotype clusters obtained in our "in-house" Tag-array minisequencing system. For each scatter plot, the mean of the average silhouette widths for the three genotype clusters were used to define a "Silhouette score" for each SNP assay. Thus the Silhouette score condenses the cluster quality for each SNP assay into a single measure that ranges from 1.0 to -1.0. When calculating the Silhouette score, the distance between data points can be measured either in one dimension, for example on the x-axis, or in two dimension using vectors, as illustrated in Figure 1. In our Tag-array minisequencing system we used distances measured only in one dimension, along the x-axis, where the signal fraction (Signal_Allele2_/ (Signal_Allele1_+Signal_Allele2_) is plotted, since this is the major determinant for genotype assignment in our system. The logarithm of the sum of the signals from both alleles (Signal_Allele1_+Signal_Allele2_) plotted on the y-axis is only used to set the cut-off values for failed genotype calls. Figure 2 shows nine examples of SNP genotype clusters that yielded different Silhouette scores. Negative controls and assays with signals below signal cut-off level are not shown in Figure 2 since they are not included in the Silhouette score calculations.

The examples in panels E, F and G of Figure 2 illustrate how different clustering patterns can yield similar Silhouette scores. Based on the results from the scatter plots used to assign genotypes in this study, our recommendation is to accept the results from SNP assays with Silhouette scores >0.65 and to fail the whole assays if the Silhouette scores is <0.25. Individual genotype calls for assays where the Silhouette score falls between 0.25–0.65 may be accepted or failed after visual inspection. Excluding some of the outliers will then increase the Silhouette score. Our recommendations is in line with Liu et al., who have included silhouette calculations in the complex algorithm used to interpret the data from the Affymetrix 10K HuSNP hybridization microarray [9].

Here we exemplify the use of Silhouette scores by comparing the performance of the TERMIPol, Therminator, KlenThermase and ThermoSequenase DNA polymerases in the Tag-array minisequencing system [2]. Twenty-six SNPs were analyzed in both polarities in 16 DNA samples in two independent experiments. As our Tag-array genotyping system utilizes an "array of arrays" format [10] with 80 subarrays on each microscope slide, we were able to test all four enzymes in all samples on the same slide at exactly the same conditions, to facilitate a fair comparison between the enzymes.

**Figure 3:**
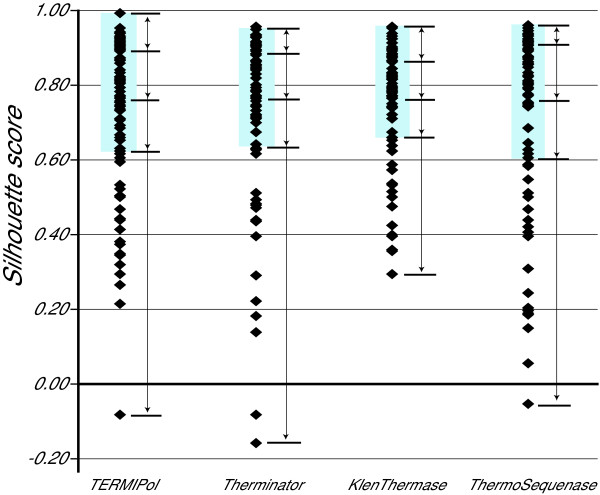
**Distribution of Silhouette scores from minisequencing assays using four DNA polymerases. **The Silhouette score is given on the y-axis. Each black diamond represents the Silhouette score for one SNP assay. The light blue rectangular boxes indicate those 75% of the scatter plots that yielded the highest silhouette scores for each enzyme. Quartiles are indicated by the black horizontal lines.

Figure 3 shows the distributions of Silhouette scores in these SNP assays. For all enzymes, 75% of the scatter plots (indicated by light blue rectangles in Figure 3) yielded silhouette scores above or close to our recommended limit of 0.65. Results from a total of 79 scatter plots/SNP assays are included in Figure 3 and Table 1. If a SNP assay failed for all samples with one enzyme, the results from this assay were excluded from the whole enzyme comparison. It should also be noted that a non-stringent genotype calling strategy was applied to reveal possible differences between the enzymes both in clustering properties and genotyping results. This is the reason for the very low Silhouette scores for some SNP assays, which normally would be considered as failed. Using 0.65 as cut-off, 70–76% of the SNP assays would have been successful in this study.

**Table 1:** Silhouette scores, signal to noise ratios and genotyping performance for four DNA polymerases in Tag-array minisequencing^1^

	Silhouette score ^2^				S/N ^3^			Genotype calls ^4^			
	
	Average	Median	Highest		Average	Highest		Correct		Errors	
			n	%		n	%	n	%	n	%

TERMIPol	0.72	0.78	20	25.3	4.3	11	13.9	2337	98.9	18	0.8
Therminator	0.69	0.79	15	19.0	3.6	7	8.9	2323	98.3	32	1.4
KlenThermase	0.74	0.79	22	27.8	8.0	21	26.6	2346	99.3	10	0.4
ThermoSequenase	0.71	0.82	22	27.8	8.9	40	50.6	2324	98.3	34	1.4

^1 ^Duplicate experiments, each with duplicate SNP assays in both DNA polarities, were performed and the results are composite values from both experiments.

^2 ^The Silhouette scores were calculated as described in Figure 1. The average and the median score for all SNPs are given for each enzyme together with the number of SNP assays (n) and frequency (%) where an enzyme yielded the highest Silhouette score.

^3 ^Signal to noise ratios (S/N) were calculated from each spot by dividing the fluorescence intensity values from the fluorescently labelled ddNTP/ddNTPs corresponding to a true genotype (signal) by the fluorescent intensity value from the other ddNTPs (noise). The average S/N ratios are given together with the number of SNP assays (n) and frequency (%) where an enzyme yielded the highest S/N.

^4 ^Number of genotype calls (n) and call rate (%). The genotype obtained from the majority of the assays was considered to be the correct one. The percentages of the samples not accounted for in the table failed to give genotypes.

In the comparison between the enzymes, KlenThermase displayed the highest average Silhouette score, ThermoSequenase had the highest median Silhouette score and also obtained the highest Silhouette score most frequently (Table 1). In addition to the Silhouettes scores, that represent a measure of the robustness of a SNP assay, the signal to noise ratios (S/N) and the genotyping success was assessed (Table 1). All four enzymes performed satisfactorily in our minisequencing assay taking into account the non-stringent genotyping criteria used. However, performance varied between the evaluated features with high error rates for Therminator and ThermoSequenase. KlenThermase showed the best results over all and, also taking into account the cost, would be the enzyme of choice based on the results from this study.

## Conclusion

We conclude that "Silhouette scores" for assessing the cluster quality is well suited for comparing the performance of SNP assays. Here we used a one-dimensional calculation of the Silhouette scores, by measuring the distances between the data-points along the x-axis only. A two-dimensional Silhouette calculation using vectors should be applied when genotypes are assigned by scatter plots with the fluorescence signals corresponding to the two alleles on the y- and x-axis. Both options are available in the ClusterA program that also calculates mean, variance and F-statistic for the input data set. The program is freely available through our website . We believe that the ClusterA program for calculating Silhouette scores created in the present study is a useful and general tool for any genotyping system, where the genotypes are called by cluster analysis with the aid of scatter plots.

## Methods

### DNA samples

Genomic DNA was extracted from blood samples from 16 volunteer blood donors using the Wizard genomic DNA purification kit (Promega, Madison, WI).

### Genotyping procedure

Twenty-six SNPs, selected to be located in unique PCR amplicons, were included in the test panel. For information on the single nucleotide polymorphisms and oligonucleotides used, see the Additional file 1: SNPinformation.pdf. PCR primers were designed and combined in multiplex PCR reactions. Minisequencing primers with 20 bp 5'-Tag sequences were designed for both DNA polarities. The experimental details of the genotyping procedure have been described in detail previously [11]. In short it included the following steps: The regions containing the sequence variations were amplified in six optimized multiplex PCRs. For each sample the PCR products were pooled and divided into four aliquots, one for each enzyme. The remaining dNTPs and primers from the PCR reaction mixture were removed by treatment with Exonuclease I and shrimp alkaline phosphatase. The cyclic minisequencing reactions were performed in solution as described below, and the extended minisequencing primers were hybridized to microarrays carrying immobilized covalently coupled oligonucleotides (cTags) complementary to the Tag-sequences of the minisequencing primers. The cTags had been immobilized to CodeLinkTM Activated Slides (Amersham Biosciences, Uppsala, Sweden) via their 3'-end NH_2_-groups to form 80 subarrays per slide, each with 60 cTags as duplicate spots. Finally the microarray slides were scanned, and the fluorescent signals were measured.

### Minisequencing reaction

Cyclic minisequencing reactions were performed in solution with 10 nM of each of the 52 tagged minisequencing primers using 0.1 μM ddATP-Texas Red, ddCTP-Tamra and ddGTP-R110 and 0.15 μM ddUTP-Cy5 (Perkin-Elmer Life Sciences, Boston, MA), and 0.064 U/μl of one of the four DNA polymerases in 15μl of 0.02% Triton-X, 4.1 mM MgCl2 and 33.6 mM Tris-HCl pH 9.5. The cyclic extension reactions were performed on a Thermal Cycler PTC-225 (MJ Research, Watertown, MA) with an initial 96°C for 3 min followed by 55 cycles of 95°C and 55°C for 20 s each. The DNA polymerases were; TERMIPol (Solis BioDyne, Tartu, Estonia), Therminator (New England BioLabs Inc., Beverly, MA, USA), KlenThermase (Gene Craft, Lüdinghausen, Germany), or ThermoSequenase (Amersham Biosciences, Uppsala, Sweden). A custom made reaction rack holding the arrayed slides with a silicon grid to give 80 separate reaction chambers was used during capture of the minisequencing reaction products on the Tag-arrays.

### Data analysis and genotype assignment

The fluorescence signals were measured from the microarray slides using a ScanArray Express^® ^instrument (Perkin-Elmer Life Sciences, Boston, MA). The excitation lasers were: Blue Argon 488 nm for R110; Green HeNe 543.8 nm for Tamra; Yellow HeNe 594 nm for Texas Red and Red HeNe 632.8 nm for Cy5. The fluorescence signal intensities were determined using the QuantArray^®^analysis 3.1 software (Perkin-Elmer Life Sciences, Boston, MA). The QuantArray file was exported to the SNPSnapper v4.0 software ) for genotype assignment. Raw data as fluorescence signals and signal ratios are provided as supplementary material, see Additional file 2: Rawdata.txt. Genotypes were assigned based on scatter plots with the logarithm of the sum of both fluorescence signals (Signal_Allele1_+Signal_Allele2_) plotted on the y-axis, and the fluorescence signal fraction, obtained by dividing the fluorescence signals from one allele by the sum of the fluorescence signal from both SNP alleles (Signal_Allele2_/ (Signal_Allele1_+Signal_Allele2_), on the x-axis [11]. The result file with the assigned genotypes and the corresponding signal ratios were exported as a text file and used to calculate Silhouettes scores using the ClusterA program. ClusterA is implemented in Microsoft Visual Basic 6.0, and can be run on PCs with the Microsoft Windows operating system. The ClusterA program also provides the mean, variance and F-statistic for the input data.

## Authors' contributions

LL planned the experiments, guided the laboratory work and performed the analysis of results, interpreted the data and drafted the manuscript. AA carried out the laboratory work and part of the data analysis and provided input to the manuscript. MJ programmed the ClusterA program and took part in the interpretation of Silhouettes. ACS initiated the study, supervised it, and coordinated the manuscript writing process. All authors have read and approved the final manuscript.

## Supplementary Material

Additional File 1 Lists the dbSNP identification numbers and the sequences of the PCR and minisequencing primers.
Click here for file

Additional File 2 Includes the raw fluorescence signals and the fluorescence signal intensity ratios for the two experiments as a tab delimited text file.
Click here for file
