# Supplementary material for: Silhouette scores for assessment of SNP genotype clusters
Source: BMC Genomics. 2005 Mar 10;6:35. doi: 10.1186/1471-2164-6-35 (PMC555759; doi:10.1186/1471-2164-6-35)
Supplement: Additional File 1 — Lists the dbSNP identification numbers and the sequences of the PCR and minisequencing primers. [file 1471-2164-6-35-S1.pdf]

Single nucleotide polymorphisms and oligonucleotides used.

dbSNP ID [1]	Variation	PCR primer Sequence 5'-3' [2]	MS-primer Sequence 5'-3' [3]
rs715405 [E]	A/G	AGGTATTACTCTGCTTACCACA<br>CTGAGATGTTATTTGCTAGTTCA	ATGCCAGGTCGTAGTCGAATAAAGACTGCTCACTGACAAAG<br>TAGTCAGGTTGGATGTCTACCAGGCATTATATTTAGGCTGA
rs5707 [D]	T/G	GCACAATGGAACAGAACTCAC<br>GAGAGGCCTGGGGACAGAA	CGATATAATGCTCGATACGGCACCGTAAGTTGGGCCGCCC<br>CAATGATAGGCTAGTCTCGCGTCCGGGGCAGATGACCT
rs383452 [D]	G/A	AACCTCTGAGACCCTGAGACAC<br>CCTTATGGTGCTGGGCACA	ATTCGCGCACCTGAGGATTTCTCCTCCTTGATAGAATGTC<br>ATTCACCTGACTCGCGTATAGCACAGTATAAAACTCACTAAA
rs715710 [D]	C/G	CACCTTCTTGTTGTATCCCCA<br>GGAGTAATCAGGTCATGAGCA	CTGACTAATCGTGTAGGTGTGAGTAGAGAGCAAGATCAAAC<br>CTAATACATCGTGATACGGGCCTTATAAGAAGAGATGCAAG
rs5186 [B]	A/C	CCCCAAAAGCCAAATCCCAC<br>AGAAAAGTCGGTTCAGTCCAC	ATGATGTGCAAAGTGCCGTCCACTTCACTACCAAATGAGC<br>CTGGACCTTAATCGTGTGCGCCTTCAATTCTGAAAAGTAGCTAA
rs714825 [C]	T/C	GGTGTCTCCATTATGCTATCCA<br>CCAGACTGTCACTTCATATTCTCA	AGCAGCATCGTAGAGCGTCATGTACATGAGGACTGAGAAAC<br>AGCTGCATCCTAGCGTCATGCCTGATTAGTTAGTTGAATAAGT
rs5333 [A]	T/C	CGTCGAGAAGTGGCAAAAACA<br>TACACAGGATCATACCTAAGTAA	ACAGGCGCTGCAACCGATATCTTTGCTGGTTCCCTCTTCA<br>AGAGCCCTGCAACGCGATATGTTTTCTTCAATATACGGCTTAA
rs5335 [B]	G/C	CAGTGGAAGAACCACGATCA<br>TCTTGGGTGTGGGAGTGAA	AATCTATCTACAAGCCGGTCAAAATCACAAGGCAACTGTGA<br>AAATGATCTCACCGCGTACTTCAGAGAAGAGATTCCCGGA
rs714932 [E]	G/A	CAAATCCCAAAAACTTCACCAA<br>TTCAAAGTCTTCAGGATAGCAA	AGTCATAGTACCTCATGCTCTCTTCTATCTCACCAGAAAAAA<br>ACTGTCGTCAACGTCAGGCAGGATAGCAAATTCTAGTGTTTTT
rs1042717 [B]	G/A	GTCACCAACTACTTCATCACTTCAC<br>TGGCAAAGTAGCGATCCAC	CTCAACAGTGTATCCGGTGGGCTGATCTGGTCATGGGCCT<br>CTGCGTACTGTATAAGGATGGCCCCAAAGGGCACCACTGC
rs1042719 [A]	G/C	GATTTCAGGATTGCCTTCCA<br>ATGGCCCACAAAGTCTTCC	CTCATTAGTATCATCGTCGGGTCTTCTTTGAAGGCCTATGG<br>GCGTTATTCAGATCGAGTGACCGTTGCTGGAGTAGCCATT
rs714470 [F]	A/C	CCTTTGAGTCACATCATTTTCA<br>AGTTAGATGTACAAGGCTGAC	ACCGTAGCTCTCGGCAAGATCGAATAGCACATGTGTAAAAAT<br>ACGCTAATGACGGCAGTGCACAAAATCTGGGAGGATAGATG
rs1799998 [D]	T/C	AATGAACTAAATCTGTGGTATAAAA<br>CCAGGGCTGAGAGGAGTAAA	GTATGTGCTGTTATCGAAGCCTATTAAAAGAATCCAAGGC<br>CTATGGCTGAGTATGTAACGCTTATCGTGAGATGAGAGGG
rs713503 [E]	G/T	AGCCTGCTACCACTTTGCCA<br>AGAGGGAGAATCAGCCTACA	GAATAGTGTCGTAGGCTCTCGGACCATGCCTTGCTTTTGT<br>GGGCACGTTGCTAAACTGTTTGCTTGAAGGAAACACGGGA
rs5092 [A]	A/G	TGTTACCACCCAGGAGCCA<br>CACCACACACTGTAGTCCC	TCGAGCGATTTATACATCCGAGTGCTGACCAGGTGGCCAC<br>CAATGATCGACCTGGAGTCTCTGAAGTAGTCCCACATCAC
rs715447 [F]	G/C	ATATCCATTTGCTCCAGCATCA<br>AAGAATAAAGTGGGAGTAATCAC	AGCATATAGGGTACGCTAGATGATGATTGTAGCTGTATTGTA<br>ATGAAGACTCGTCCCTCGATTCTACCTGATTGATACTAAAAC
rs13841 [F]	C/T	AGGAAAGAGAGCACCTGCCA<br>GGAGAATCCTTCAAAATGCTAC	ACCATATAGCCATCTAAGCGATCACTCAATCATTTTTCTGGA<br>CCTCGATATGTACGGATAGGCCATTTCTAGCTGTTAACCCT
rs6083 [F]	A/G	CCCTTACCCCTGCTTTCCC<br>CTGTTTGATGCCCACGCTCA	CAGCCGAATGGACTATCGCTTCTTTCTCCAGATGATGCCA<br>TAGTTGACTTGGAGTATGCCTATGAATGGCATCCACAAAA

rs715296 [C]	G/C	GTTTTACCATAGACGGATTTACA<br>CTTAATGTCTAATGGATGCAAAC	CCAGCTCGTACTATCGAATAATTTTGGTTAATATCTTAATACCA<br>CACCATCGTCGTATCGAATATTAAAGAGTATAATGCCAAAAGA
rs5882 [D]	A/G	TGCTCCAGGGAGGACTCAC<br>CAGCCCACACTTACGAGACA	ACAGTCGCCTAGCGGTATATTGCAGAGCAGCTCCGAGTCC<br>AGGTACGCTCCTAGTCCATAGACTGCAGGAAGCTCTGGA
rs713197 [E]	G/T	AATCGATATTTTGATCCATTGAC<br>CTCTTTGCCTTGAACCTTGAC	GATAAGCGTTCACAGCTCGGTGGGGTCATAAGATTTCTTCAA<br>CTCAAATCTTAGCACTCGTGAATATTTAAAAAATGTACCAAAATAT
rs1578401 [A]	A/G	TTTCCTGATGCTACAGACCAC<br>AACCTGGGATAATAAATACAGAC	GGCTACTATTCGATCATACGAATGCCATCACTTTTCCTCATT<br>CGCCTATCTGGTAATAACTGCCATATTTACTTATATACCTGAAA
rs753381 [C]	C/T	CCCAGGTTGGCAGTGGCA<br>GCACACAGGAGACAAGCACA	CGTGACAATGTACTATGGAGGAGGACGAGCTGACCTTCA<br>CGCGTCGATCTAGGTGACTACTGGATGATGGCGCTCTTG
rs715147 [F]	A/G	GACAGCCATAGAATTCCAGCA<br>CTTGACCTGTCTTTGAAGCCA	GGTGATACTTCGCTATATGGCTTAGAAACTAGCCAGCCCT<br>GGCTATACTTGACAGTGATGGTTGCCTTGTCTGCCAGTG
rs713625 [F]	C/T	TCTGCTAGGAAATCTCAGAAC<br>GCTCAACACTGCGAGGTCA	GGTTCCTCTGATGTGCTAGACTTTCTCCAGCATAGCAAAAG<br>CAGTTCTACGATGGCAAGTCTCTCCTGCCTCTGGGGAT
rs713753 [E]	C/T	TAGAGGCAACTCCAGGTCAA<br>TTGGTGCCTCTCTTTCCACA	GCGGTATGTCGTGCTATAATGTCAAAGGTGGGGAACCAA<br>GGCGTCTGTCGTGCTCATAACTTTGGGCAGGATCTTGGT

[1] The identification numbers are from the dbSNP database: http://www.ncbi.nlm.nih.gov/SNP/. The indexes (A-F) indicate which SNPs has been amplified together in multiplex PCR.

[2] PCR primer sequences. The forward primers are given above their corresponding reverse primers. See further information in Lovmar, L. et al. (2003) *Nucleic Acids Res*, **31,** e129.

[3] Minisequencing primers. The primers with the same polarity as the forward PCR primers are given above the primer on the complementary strand. The 20 most 5' bases of the primer are tag-sequences with complementary cTags included in the printed arrays.
